# Long-Term Phytaspase Responses in *Nicotiana benthamiana*: Sustained Activation by Mechanical Wounding, but Not by Drought, Heat, Cold, or Salinity Stress

**DOI:** 10.3390/ijms26157170

**Published:** 2025-07-24

**Authors:** Maria Alievna Abdullina, Jiarui Li, Feifan Liu, Xinyi Luo, Anastasia Igorevna Barsukova, Svetlana Vladimirovna Trusova

**Affiliations:** 1 Faculty of Biology, Shenzhen MSU-BIT University, 1 International University Park Road, Dayun New Town, Longgang District, Shenzhen 518172, China; maria.lenpack@gmail.com (M.A.A.); 1120220412@smbu.edu.cn (J.L.); 1120220380@smbu.edu.cn (F.L.); 1120230462@smbu.edu.cn (X.L.); 2 Faculty of Chemistry, Shenzhen MSU-BIT University, 1 International University Park Road, Dayun New Town, Longgang District, Shenzhen 518172, China

**Keywords:** subtilase, phytaspase, *Nicotiana benthamiana*, plant stress responses, wounding, drought, long-term responses, enzyme activity, gene expression

## Abstract

Plant subtilases, as hydrolytic enzymes, contribute to certain plant stress response pathways by cleaving precursor proteins into active peptides or through other less well-characterized mechanisms. Phytaspases represent a specific subgroup of subtilases, and their participation in rapid stress responses, particularly to herbivory attacks and drought, is already well established, in contrast to their poorly understood role in long-term responses. This study investigated the involvement of phytaspase NbSBT1.9-2 in the long-term stress responses of *Nicotiana benthamiana*. Plants were subjected to either mild to severe mechanical wounding or drought stress, followed by the detection of phytaspase activity and gene expression in the leaf tissue. The results revealed a distinct involvement of phytaspase in the wounding response, showing increased activity and upregulated expression correlated with the extent and recurrence of wounding. In contrast, no significant change in phytaspase activity was observed in the leaves under drought, alongside salinity and heat stress conditions. Consequently, phytaspase association with the long-term response to mechanical injury was demonstrated using *N. benthamiana* as a model organism.

## 1. Introduction

Plants’ limited mobility has necessitated the evolution of sophisticated molecular mechanisms for defense and adaptation to cope with adverse environmental conditions, including biotic and abiotic stresses [1,2,3]. The first line of defense for plant cells is the cell wall, with its soluble fraction, the apoplast, serving as a critical interface for these protective responses [4,5]. Within the apoplast, a substantial portion of its high proteolytic activity is mediated by subtilisin-like proteases (subtilases), a family of serine proteases predominantly localized in the apoplast [6,7,8]. Subtilases are thought to play a pivotal role in plant defense by facilitating the activation of molecular signaling pathways through the hydrolysis of protein precursors, thereby releasing mature bioactive peptides that orchestrate stress responses. Subtilases directly cleave peptide precursors to produce functional signaling molecules, and also indirectly modulate downstream signaling cascades that enhance plant resilience [8,9,10,11]. Among subtilases, phytaspases, with caspase-like specificity for aspartic acid residues, contribute to stress-induced signaling, programmed cell death (PCD), and immune responses, underscoring their multifaceted roles in adaptation [12,13,14,15]. Overall, subtilases are recognized for playing an important and multifaceted role in plant defense responses against diverse biotic challenges (such as pathogens, herbivorous insects, and other animals) and abiotic stresses (e.g., salinity, drought, waterlogging, and heat stress), although their precise molecular mechanisms are not fully elucidated.

Beyond subtilases, other stress-responsive proteases, such as vacuolar processing enzymes (VPEs) and papain-like cysteine proteases (PLCPs), also participate in programmed cell death and adaptive responses [16,17,18,19,20,21]. While phytaspases exhibit strict substrate specificity toward aspartic acid residues in the P1 position, PLCPs show broader specificity, preferentially recognizing hydrophobic residues such as leucine, valine, or alanine, though some may target aspartate [22]. VPEs, in turn, cleave after asparagine and aspartate [23], suggesting potential functional interplay in proteolytic signaling networks.

Plant responses to stress-inducing conditions manifest as rapid responses that emerge within hours to mitigate immediate threats, alongside long-term responses that persist over extended periods to support sustained adaptation to ongoing environmental pressures. Rapid responses encompass the activation of defense signaling pathways, such as the proteolytic processing of pre-synthesized peptides to quickly generate signaling molecules during pathogen attack, and also include calcium-mediated signaling in response to stresses such as osmotic stress, salinity, or cold [24,25]. In contrast, long-term responses facilitate the gradual adjustment of metabolic and genetic processes to endure chronic challenges such as drought and salinity, including the accumulation of osmoprotectants like proline to maintain cellular water balance, the repair of photosystem II through FtsH protease activity, and the processing of immunomodulatory peptides like PEP1 to sustain immune defense [26,27,28].

Subtilases play a decisive role in orchestrating rapid plant responses by processing pre-existing peptides into active signaling molecules. For example, the *Arabidopsis thaliana* subtilase SBT3.3 (AT1G32960) rapidly cleaves extracellular substrates, triggering salicylic acid (SA)-dependent immune priming within hours to enhance defense against microbial invasion [24]. In *Solanum lycopersicum* (tomato), the phytaspase SlSBT3 hydrolyzes prosystemin to produce systemin [29], thereby activating the jasmonate (JA) pathway to synthesize peptide inhibitors against herbivore attack [30,31]. This pathway mediates resistance to salinity in *A. thaliana* [32]. The jasmonate pathway regulates resistance in *Panax notoginseng* against the root rot pathogen *Fusarium* [33]. It additionally mediates resistance in *Nicotiana tabacum* against the pathogen *Phytophthora nicotianae*, but the details of subtilase involvement in these cases remain scarce [34]. Plants lacking systemin rely on alternative regulatory peptides that activate the JA signaling cascade [35], and subtilase involvement in these processes is proposed.

Additionally, the *A. thaliana* subtilase AtS1P (AtSBT6.1, AT5G19660) participates in the processing of the peptide hormone AtRALF23 precursor, recognizing the RRIL site, which highlights the role of subtilases in the maturation of signaling molecules. Moreover, AtS1P is suggested to contribute to the maturation of pectin methylesterase by cleaving its proprotein, thus aiding plant defense against fungal infections by strengthening cell walls [10].

Another regulatory pathway for stress responses involves phytosulfokine, a peptide hormone that modulates plant adaptation to drought. This rapid response, driven by calcium-dependent pathways, transitions under prolonged stress into long-term reactions, such as oxidative stress and metabolic restructuring [36,37]. In *S. lycopersicum*, the phytosulfokine precursor is hydrolyzed by phytaspase 2, producing mature phytosulfokine peptides that mediate flower and fruit abscission under drought conditions, accompanied by an increased expression of phytaspase 2 in pedicel tissues [14]. In *A. thaliana*, the subtilase SBT3.8 (AT4G10540) hydrolyzes the phytosulfokine precursor, enhancing drought tolerance through the regulation of stress-responsive pathways. Notably, the expression of SBT3.8, along with SBT1.4 and SBT3.7, is upregulated in roots under drought-imitating D-mannitol treatment, promoting lateral root development and contributing to improved stress resilience [11]. SBT3.8 subtilase has been characterized as the only phytaspase of *A. thaliana* [38].

Plant defense responses to drought, salt, and cold stresses can be mediated by BURP-domain peptides activated by the hydrolysis of their precursors [39,40], and subtilases are thought to contribute to these processes. Although direct evidence for the role of subtilases in processing BURP-domain precursors has not yet been described, indirect evidence supports their involvement in BURP-peptide signaling. In *A. thaliana uspl1* mutants lacking the BURP-domain protein AtUSPL1, the subtilase gene *AtSBT5.3* (At4G10550) exhibited a 23-fold increase in expression under normal conditions, along with 17 other genes, 5 of which are associated with stress responses [41]. This suggests a potential role for AtSBT5.3 in stress-related signaling.

Subtilases are also involved in stress response pathways through the regulation of transcription factor activity. In *A. thaliana*, subtilase AtS1P (AtSBT6.1, AT5G19660) is a key component of the salt stress response pathway, facilitating the proteolytic release of the membrane-associated transcription factor AtbZIP17 from the endoplasmic reticulum. This enables AtbZIP17 translocation to the nucleus and the activation of salt stress-responsive genes [10]. A rare example is the alternatively spliced barley subtilase SBT5.2(b), which lacks a signal peptide and is retained on intracellular vesicle surfaces via N-terminal myristoylation, interacting with and sequestering the defense-related transcription factor MYB30 to modulate immune responses [42]. These findings highlight diverse subtilase-mediated mechanisms in regulating stress-responsive transcription factors.

Another rare pathway involves the direct release of a regulatory peptide from the protease-associated (PA) domain of a subtilase. The only described example is GmSubPep, isolated from *Glycine max* leaves, which activates plant defense mechanisms [43]. GmSubPep is embedded within a subtilisin-like proteaseencoded by the gene *Glyma18g48580* and functions as a damage-associated molecular pattern (DAMP), enabling plants to recognize tissue damage and initiate immune responses. It induces the expression of defense-related genes, such as Cyp93A1, Chib-1b, and PDR12, in response to pathogen attack or mechanical wound [44].

Collectively, subtilases, including defense-specialized enzymes such as phytaspases, mediate the rapid responses to diverse stress conditions. Although phytaspase was initially identified as a participant in PCD in response to viral infection, the identification of specific phytaspase substrates reveals its broader significance in stress responses. In addition to the previously characterized substrates prosystemin, associated with wounding stress, and phytosulfokine, linked to drought stress, calreticulin has been identified as a substrate of tobacco phytaspase [45]. Calreticulin, a calcium-binding molecular chaperone [46], participates in calcium-mediated signaling pathways triggered by certain biotic and abiotic stresses [47]. Phytaspase involvement in calreticulin reticular localization disruption indirectly underscores the protective and regulatory role of phytaspases in stress response development. Such a substrate repertoire suggests that phytaspase activity may be influenced by specific stress-induced signaling cascades. The activation of prosystemin in tomato directly implicates phytaspases in the JA pathway, which is typically induced by wounding. Phytosulfokine and calreticulin point to the involvement of calcium-mediated signaling, which is common in responses to both abiotic and biotic stress. While no direct link has been established between phytaspases and abscisic acid (ABA) or salicylic acid (SA) signaling, a modulatory effect through these hormonal pathways cannot be excluded.

Furthermore, efforts to identify the substrates or interacting partners of tobacco phytaspase during PCD have revealed a tubby-like F-box 8 protein [48], which is intriguingly implicated in the clathrin-mediated internalization of phytaspase during the development of PCD triggered by oxidative stress, a process described for these particular subtilases in tobacco [13,49], *A. thaliana* [38,50], and *Nicotiana benthamiana* plants [51]. The identification of phytaspase substrates and protein partners remains a challenge. In *N. benthamiana*, a plant lacking systemin, the involvement of other peptides in adaptive responses, potentially processed by phytaspases or other subtilases, can be hypothesized. However, these questions remain among the unsolved mysteries, and exploring the conditions of phytaspase activation in response to stimuli represents a promising avenue for discovering new regulatory peptides. The role of phytaspases in sustaining long-term adaptive responses to prolonged or intense stress is supported by indirect evidence but remains poorly understood, necessitating further investigation. Here, we demonstrate the impact of sustained stresses, such as wounding, drought, heat, and salinity, on the activity of a recently characterized *N. benthamiana* phytaspase [51]. Our findings suggest the involvement of this phytaspase in long-term defense responses, at least in response to wounding. This further highlights that the role of phytaspases extends beyond merely executing programmed cell death, encompassing a protective function that contributes to plant survival.

## 2. Results

### 2.1. Severe Wounding Induces Sustained Increase in Phytaspase Activity

**Figure 1:**
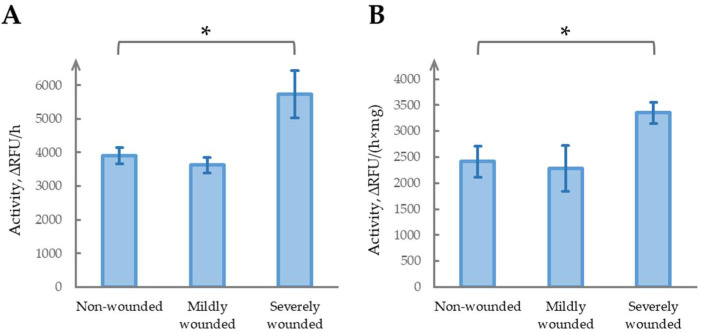
Comparison of phytaspase activity in four-week-old *N. benthamiana* leaves 2 weeks after mild (gently damaging leaf lamina) and severe (removal of five mature leaves) wounding. Data represent means ± SD from three biological replicates, * indicates a statistically significant difference (*p* < 0.05, independent samples *t*-test). The coefficients of variation (CV) of non-wounded, mildly wounded, and severely wounded groups are all below the threshold value of 20%, indicating acceptable variability within the measurements. (**A**). ∆RFU/h, relative fluorescent units per hour. (**B**). ∆RFU/(h×mg), relative fluorescent units per hour per mg of total protein.

To evaluate the long-term effects of simulated herbivory, we established three experimental groups: mildly wounded, severely wounded, and undamaged control plants. Four-week-old *N. benthamiana* plants at the five-mature-leaf stage were used. Mild wounding was performed by gently damaging the leaf lamina (as described in [52]), while severe wounding involved removing four leaves, leaving one mature leaf and one to two underdeveloped leaves. The plants were maintained in a climate chamber for two weeks before sampling and the analysis of phytaspase activity (Figure 1). Mild wounding did not significantly alter long-term phytaspase activity compared with the control (*p* = 0.22, *t*-test). In contrast, severe wounding resulted in a pronounced and significant 1.47-fold increase in phytaspase activity (*p* < 0.05). These results suggest that severe wounding may induce sustained changes in phytaspase activity, whereas mild wounding does not.

To verify that straightforward normalization to fresh leaf mass provides consistent results, we additionally measured the total protein concentration in the tissue extracts and normalized phytaspase activity accordingly (Figure 1B). Although the absolute activity values differed slightly between the two approaches, the overall trend remained unchanged: no significant change in mildly wounded plants (*p* = 0.70), and a statistically significant elevation in severely wounded samples (*p* < 0.05). The magnitude of the effect was 1.47 ± 0.28-fold based on fresh weight, and 1.39 ± 0.26-fold when normalized to total protein content, indicating a negligible impact of the normalization strategy. Therefore, we continued using fresh weight normalization in subsequent experiments, although protein-based correction was occasionally applied for internal validation.

### 2.2. Time-Dependent Increase in Phytaspase Activity Following Severe Wounding

**Figure 2:**
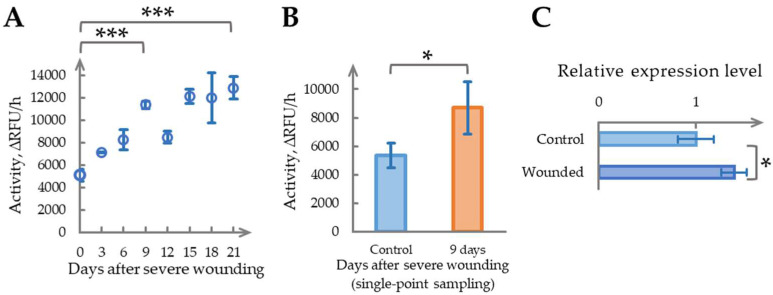
Time-dependent determination of phytaspase activity and gene expression following severe wounding in six-week-old plants. (**A**). Phytaspase activity in *N. benthamiana* leaves after severe wounding (removal of five mature leaves) measured sequentially from Day 0 to Day 21. ∆RFU/h, relative fluorescent units per hour. (**B**). Phytaspase activity in *N. benthamiana* leaves on Day 9 after severe wounding (removal of five mature leaves) with single-point sampling, across 3 independent batches. ∆RFU/h, relative fluorescent units per hour. (**C**). Phytaspase *NbSBT1.9-2* gene expression in *N. benthamiana* leaves on Day 9 after severe wounding (removal of five mature leaves) with single-point sampling. Data represent means ± SD from three biological replicates. * indicates *p* < 0.05, *** indicates *p* < 0.001. Sample with CV above threshold value of 20% is marked in a shade of red.

To investigate the temporal dynamics of the pronounced increase in phytaspase activity observed after severe wounding, we examined its time course in *N. benthamiana* leaves. Six-week-old plants were used, which had developed one additional mature leaf compared with the previous experiment. Severe wounding was induced by removing five mature leaves, leaving one nearly full-sized (uppermost) leaf and two developing leaves intact. The samples were collected sequentially from the same plants: the “Day 0” control represented tissue from the removed leaves immediately after wounding, while the samples for Days 3, 6, and up to Day 21 were obtained from the remaining leaves. The phytaspase activity measurements are presented in Figure 2A. Severe wounding significantly elevated phytaspase activity, displaying a near-linear increase from Day 0 to Day 9. On Day 9, the activity reached a 2.23 ± 0.29-fold increase relative to Day 0 (*p* < 0.01). After Day 9, the temporal patterns became more variable: a decline on Day 12 was followed by a gradual rise through Days 15 to 21. By Day 21, the activity showed a 2.52 ± 0.46-fold elevation compared with the baseline (*p* < 0.001), suggesting a slow plateauing trend.

Compared with the experiment in Section 2.1, sequential sampling resulted in a greater overall increase in phytaspase activity: a 2.38 ± 0.37-fold elevation relative to the control on Day 15, versus a 1.47 ± 0.28-fold increase observed on Day 14 with single-point sampling. We hypothesized that repeated sampling, involving additional mechanical disturbance to the leaf lamina, might enhance phytaspase activation. To test this assumption, a separate severe wounding experiment was conducted with single-point sampling on Day 9, corresponding to the peak observed in the sequential time course. As shown in Figure 2B, phytaspase activity still increased significantly following a single severe wounding event, reaching 1.62 ± 0.60-fold on Day 9 (*p* < 0.05). However, this response was weaker than the 2.23 ± 0.29-fold increase observed under repeated sampling conditions over the same period. These findings suggest that recurrent sampling in wounded plants may amplify phytaspase activation. By comparison, in Section 2.1, where single-point sampling was performed two weeks post treatment, phytaspase activity showed a 1.47 ± 0.28-fold elevation, which is similar in magnitude to the Day 9 single sample response, and indicative of a typical outcome following a single severe wounding event.

Additionally, in the single-point sampling experiment on Day 9, phytaspase gene expression was analyzed (Figure 2C). The transcript levels increased by 1.28 ± 0.26-fold, aligning with the enzymatic activity rise (*p* < 0.05). Notably, both phytaspase activity and gene expression returned to baseline by Day 21, in the absence of further mechanical damage.

These findings indicate that severe wounding induces a time-dependent increase in phytaspase activity, peaking around Day 9 and followed by post-peak fluctuations and gradual stabilization. This enzymatic response appears to correlate with increased phytaspase gene expression, with the magnitude of the activation influenced by whether wounding was single or repeated. Such dynamics may reflect a localized protective mechanism that contributes to stress resilience, and warrant further investigation, particularly in the context of sustainable adaptive responses in plants.

### 2.3. Repeated Severe Wounding Induces a Pronounced Long-Term Increase in Phytaspase Activity

To assess the effects of repeated severe wounding in *N. benthamiana*, we used plants that had previously undergone a single wounding event and whose phytaspase activity and gene expression had returned to near-baseline levels 21 days post treatment (see Section 2.2). A second severe wounding was applied by removing all six mature leaves that had developed since the initial treatment. Day 0 samples for enzyme activity analysis were collected from the excised leaves immediately after wounding.

**Figure 3:**
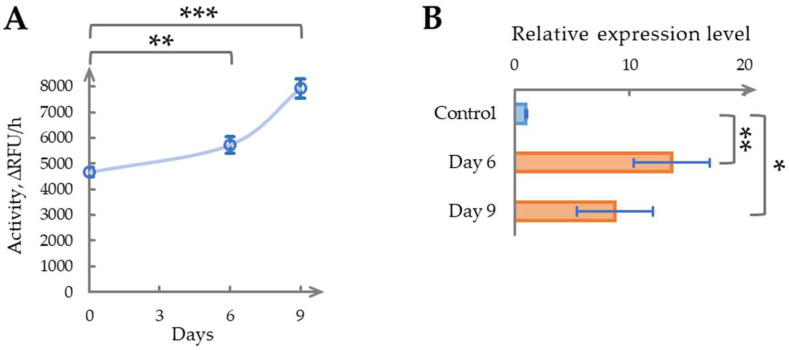
Long-term determination of phytaspase activity and relative gene expression in six-week-old plants during repeated severe wounding (removal of six mature leaves 21 days after initial wounding). (**A**). Phytaspase activity in *N. benthamiana* leaves after repeated severe wounding. ∆RFU/h, relative fluorescent units per hour. (**B**). Phytaspase relative gene expression in *N. benthamiana* leaves following repeated severe wounding (removal of six mature leaves 21 days after initial wounding) on Days 6 and 9. Data represent means ± SD from three biological replicates. * indicates *p* < 0.05, ** indicates *p* < 0.01, *** indicates *p* < 0.001. Samples with CV above threshold value of 20% are marked in a shade of red.

Given that phytaspase activity plateaued after Day 9 in earlier experiments, we hypothesized that transcript accumulation might reach its peak earlier in the time course. To test this, both the enzymatic activity and gene expression were measured on Days 6 and 9 following repeated wounding (Figure 3A,B). On Day 6, phytaspase activity increased significantly to 1.23 ± 0.12-fold relative to Day 0 (*p* < 0.01, Figure 3A), while gene expression showed a dramatic rise to 11.22 ± 3.12-fold (*p* < 0.01, Figure 3B). By Day 9, enzymatic activity further increased to 1.70 ± 0.15-fold (*p* < 0.001), surpassing the 1.47-fold effect observed with single wounding (Section 2.1), yet remaining below the 2.15-fold response recorded under sequential sampling (Section 2.2). At the same time, gene expression on Day 9, although lower than the Day 6 peak, remained substantially elevated at ~7-fold above baseline (*p* < 0.05), supporting our hypothesis of an earlier transcriptional peak.

These findings demonstrate that repeated severe wounding triggers a pronounced and sustained increase in phytaspase activity, accompanied by a marked elevation in gene expression. The sharper transcriptomic response, peaking around Day 6, suggests a stronger molecular activation under repeated stress compared with single wounding. This pattern may reflect an amplified defense mechanism in response to recurring damage, warranting further investigation.

### 2.4. Damage Severity Determines the Development of Long-Term Phytaspase Activity Effects

**Figure 4:**
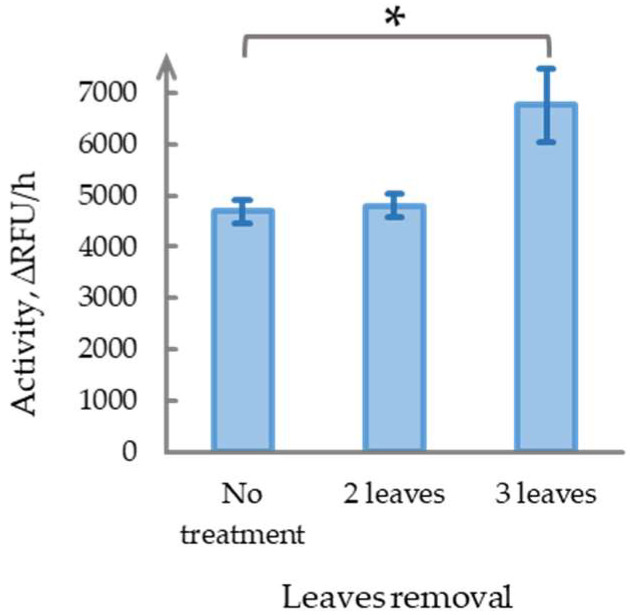
Effect of damage severity on phytaspase activity in five-week-old *N. benthamiana* leaves nine days after removal of 2 or 3 middle-tier leaves compared with undamaged controls. Data represent means ± SD from three biological replicates. ∆RFU/h, relative fluorescent units per hour. * indicates *p* < 0.05.

Given that mild mechanical stimulation failed to induce long-term changes in phytaspase activity, whereas severe damage (removal of 4–6 leaves) led to an approximate 1.6-fold increase by Day 9, we aimed to identify the damage threshold required to elicit such responses. Five-week-old *N. benthamiana* plants were used, and either two or three mature leaves from the middle tier were removed, leaving fully developed leaves above and below the damaged zone. The control plants were left undamaged. All plants were maintained in a climate chamber for nine days, after which newly formed mature leaves from the same tier were sampled for phytaspase activity analysis (Figure 4).

The removal of two leaves did not significantly affect phytaspase activity compared with the control group (*p* = 0.80), suggesting that this level of injury can be classified as mild. In contrast, the removal of three leaves led to a statistically significant increase in activity (1.44 ± 0.36-fold, *p* < 0.05), comparable to the response observed following the removal of 4–6 leaves. These results suggest the existence of a threshold damage intensity—potentially linked to the removal of more than three leaves or over half of the mature foliage—required to trigger long-term phytaspase activation. However, the exact threshold remains to be determined.

### 2.5. Drought Does Not Reliably Induce Phytaspase Activity

To assess the impact of drought stress on phytaspase activity in *N. benthamiana*, 4-week-old plants were cultivated under standard humidity conditions (~60%) and divided into two groups, well-watered controls and drought-treated plants, for which irrigation was suspended. The first signs of wilting appeared around Day 20. To prevent plant death, 10 mL of water was provided every 3 days starting from Day 20. Despite this, leaf turgor continued to decline, and by Day 34, the plants exhibited clear drought stress symptoms. Samples collected on Days 20 and 34 were used to evaluate enzyme activity, *NbSBT1.9-2* gene expression, and drought-associated metabolic changes.

**Figure 5:**
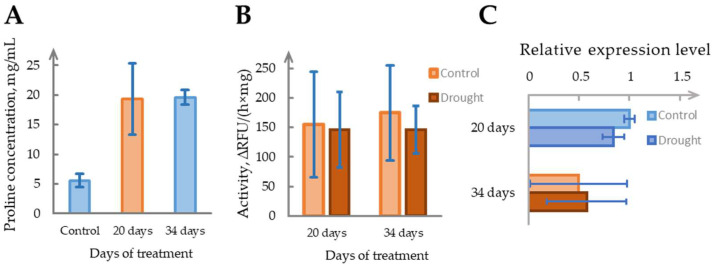
Determination of drought effect on phytaspase. (**A**). Proline concentrations as drought stress indicator of samples treated for 20 and 34 days, compared with control group, mg per mL. (**B**). Time-dependent changes in phytaspase activity in *N. benthamiana* leaves under drought stress (cessation of regular watering) compared with normally watered controls on Days 20 and 34. ∆RFU/(h×mg), relative fluorescent units per hour per mg of dry weight. (**C**). Phytaspase relative expression level in drought samples, compared with control samples, on Days 20 and 34. Data represent means ± SD from three independent batches. Samples with CV above threshold value of 20% are marked in shades of red.

Free proline accumulation, a known biochemical marker of drought stress, increased ~3.5-fold in drought-treated plants at both timepoints, confirming physiological response (Figure 5A). The soil moisture content decreased progressively, from 68 ± 10% in controls to 51 ± 7% on Day 20 and 27 ± 10% by Day 34. To account for the potential shifts in tissue water content and protein levels, phytaspase activity was normalized to dry weight. No significant difference was observed between the drought and control groups at either timepoint (Figure 5B). *NbSBT1.9-2* gene expression also failed to show consistent regulation under drought stress (Figure 5C).

Although isolated samples exhibited sporadic changes in enzyme activity or transcript levels, these effects were not reproducible across biological replicates. Based on these results, phytaspase does not appear to play a reliable role in drought adaptation under the conditions tested. However, given the observed variability and occasional induction in individual plants, we cannot exclude the possibility that specific drought scenarios or combined stress factors may elicit more robust responses.

### 2.6. Heat Stress Does Not Significantly Affect Phytaspase Activity

**Figure 6:**
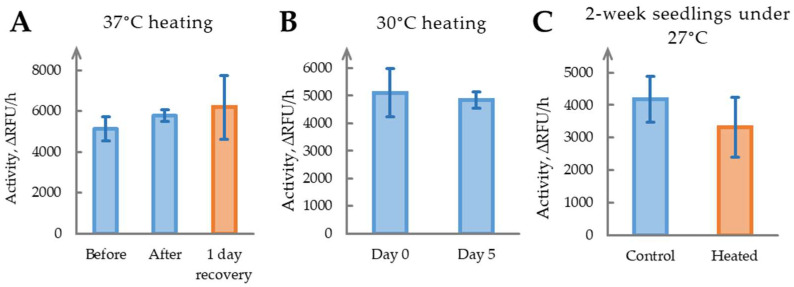
Determination of heat stress effect on phytaspase activity. (**A**). Phytaspase activity in *N. benthamiana* leaves before, immediately after, and one day after overnight exposure to 37 °C heat stress. (**B**). Phytaspase activity in *N. benthamiana* leaves after five days of 30 °C heating compared with controls. (**C**). Phytaspase activity in *N. benthamiana* seedlings after two weeks of growing under 27 °C heat stress. ∆RFU/h, relative fluorescent units per hour. Data represent means ± SD from three biological replicates. Samples with CV above threshold value of 20% are marked in a shade of red.

To evaluate the effect of heat stress on phytaspase activity, 4-week-old *N. benthamiana* plants were exposed to 37 °C overnight. Samples were collected before treatment, immediately after exposure, and following one day of recovery under optimal conditions. Post-stress symptoms included notable wilting, which persisted despite recovery measures, indicating that the imposed temperature was excessively high. Phytaspase activity measurements (Figure 6A) showed no significant changes (*p* > 0.05). Increased variability at the final timepoint suggests heterogeneous and non-reproducible responses to acute heat stress.

To assess whether a milder thermal regime could trigger phytaspase activation, we reduced stimulus intensity and extended its duration: plants were maintained at 30 °C for five consecutive days. Phytaspase activity remained unchanged between Day 0 and Day 5 (Figure 6B), and plants retained a healthy appearance throughout the experiment.

To further test heat sensitivity across developmental stages, two-week-old seedlings were cultivated at 27 °C for two weeks. Again, phytaspase activity did not differ significantly between the control and treated groups (Figure 6C), reinforcing the conclusion that the enzyme does not participate in adaptation to moderate or acute heat stress under the conditions tested.

### 2.7. The Role of Phytaspase in Salinity Stress Remains Unclear

**Figure 7:**
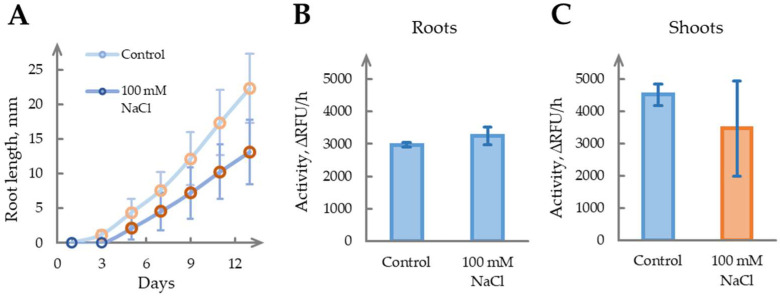
Determination of salinity stress effect on phytaspase activity in two-week-old-seedlings. (**A**). Root length of *N. benthamiana* seedlings grown on nutrient medium supplemented with 100 mM NaCl compared with control seedlings cultivated on standard medium. Light-shaded circles indicate control values for root length, while dark-shaded circles represent measurements under salinity stress (**B**). Phytaspase activity in roots of *N. benthamiana* seedlings under salinity stress. Seedlings grown on nutrient medium with 100 mM NaCl are compared with controls on standard medium. ∆RFU/h, relative fluorescent units per hour. (**C**). Phytaspase activity of *N. benthamiana*’s seedlings shoots under salinity stress (similar to (**B**)). ∆RFU/h, relative fluorescent units per hour. Samples with CV above threshold value of 20% are marked in a shade of red.

To investigate the effect of salinity stress on phytaspase activity, sterile *N. benthamiana* seedlings were cultivated on nutrient medium for 14 days with or without supplementation with 100 mM NaCl. Salt treatment had a pronounced impact on root morphology: treated roots exhibited shorter lengths compared with the controls, which is consistent with reported osmotic stress responses, including inhibited elongation and enhanced lateral branching [53] (Figure 7A). The emergence of aerial parts was delayed by ~4 days in salt-treated seedlings, but shoot development subsequently equalized, reaching the first true leaf stage approximately 1 day after the controls.

Given the differential growth dynamics, phytaspase activity was measured separately in the roots and shoots on Day 14 (Figure 7B,C). The seedling roots showed a reduced length under salinity (*p* < 0.05), but the fresh weight remained comparable between the groups (2.4 ± 0.3 mg for control and 2.8 ± 0.4 mg for treated roots, n = 10), in agreement with phytaspase activity levels, which did not significantly differ. In the shoots, salinity-treated seedlings exhibited a higher fresh weight (2.1 ± 0.3 mg vs. 1.3 ± 0.3 mg, *p* < 0.05), while phytaspase activity showed a mild but non-significant decrease compared with the controls.

Overall, no significant correlation was found between salt stress and phytaspase activity in either organ type. The physiological changes observed under salinity do not appear to be mediated by phytaspase, suggesting its limited involvement in the adaptation to ionic or osmotic stress under the tested conditions.

## 3. Discussion

Our study investigated the role of phytaspase activity and gene expression in 4–6-week-old *N. benthamiana* plants and 2-week-old seedlings under various stress conditions, including mechanical wounding, drought, heat stress, and salinity. The results reveal diverse responses to these stressors, highlighting the complexity of phytaspase regulation in plant stress responses and the variety of molecular pathways mediating stress-induced reactions.

Phytaspase was shown to contribute to the formation of long-term adaptive responses to mechanical wounding, which may mimic the effects of insect or herbivore feeding. Rapid plant responses to mechanical wounding, such as prosystemin activation and initiation of the jasmonate pathway, are well documented. Although phytaspase involvement in these processes has been previously reported [29], this study is the first to demonstrate its contribution to establishing a sustained long-term response. The molecular mechanisms underlying this regulatory pathway remain a subject for future research; however, certain novel insights were obtained.

A significant long-term increase in phytaspase activity required substantial mechanical wounding. We determined that removing three or more leaves was necessary to elicit this effect, with the response being comparable for the removal of three to six leaves. This suggests the existence of a threshold intensity of wounding. Minor damage to the leaf blade, the regular removal of small leaf portions associated with sampling for analysis, or even the removal of up to two leaves did not alter phytaspase activity. Thus, some wounds can be considered “mild”, where plants do not develop responses mediated by increased phytaspase activity. Other wounds should be classified as “severe”, and exceeding this threshold stimulates a phytaspase-related response pathway. However, the precise threshold—whether defined by the number of leaves or the proportion of mature foliage removed—remains unclear and requires further investigation. Repeated or regular wounding above this threshold may enhance phytaspase activity to varying degrees (see Section 2.2 and Section 2.3). This modulation appears to be linked with de novo enzyme production, as shown in Section 2.3. Conversely, repeated “mild” stimuli do not increase phytaspase activity, likely conserving plant resources to avoid overreacting to minor incidental damage.

The observation that a threshold exists, where removing two leaves, partial leaf removal, or damage to leaf parts did not lead to long-term changes in phytaspase activity, supports the feasibility of conducting experiments with sequential sampling without unintentionally elevating phytaspase activity due to wounding effects.

Interestingly, rapid responses to wounding, such as systemin activation, are linked to the mixing of intracellular and extracellular leaf components, enabling the processing of prosystemin by apoplastic enzymes like phytaspase. Additionally, plant signaling systems may respond to the presence of insect proteins, with response activation linked to herbivore-associated molecular patterns (HAMPs) [54]. However, the removal of three or more leaves in young *N. benthamiana* plants is perceived as a “severe” impact, despite the complete absence of insect proteins and leaf blade damage, as leaf removal occurs at the petiole level. This indicates that plants possess signaling systems capable of detecting the loss of a leaf, a certain proportion of leaves, or photosynthetic surfaces in some manner.

In contrast, despite the physiological markers of drought stress—reduced soil moisture, wilting symptoms, and significant proline accumulation—phytaspase activity did not show consistent induction under water-deficient conditions. The enzymatic measurements normalized to dry weight revealed no significant differences between the treated and control plants across two sampling points. The transcript levels of the NbSBT1.9-2 gene were variable and lacked reproducibility across biological replicates, suggesting no stable transcriptional regulation under water deficiency. While sporadic increases in activity or expression were observed in individual plants, the overall lack of consistency implies that phytaspase is not reliably engaged in drought stress signaling. This contrasts with its long-term activation following mechanical wounding and points to selective responsiveness rather than broad involvement in abiotic stress adaptation.

Heat stress also did not significantly affect phytaspase activity across various experimental conditions. Exposure to 37 °C caused wilting and heterogeneous responses but failed to enhance phytaspase activity, possibly due to excessive stress intensity. Milder conditions (30 °C for 5 days or 27 °C for 2 weeks) also showed no changes, with plants maintaining a good condition or exhibiting minimal alterations in enzyme activity. This suggests that phytaspase is unlikely to contribute to the adaptation to elevated temperatures or heat shock responses in *N. benthamiana*, contrasting with its role in molecular responses to mechanical wounding and drought.

The investigation of salt stress, applied for 2 weeks, revealed differences in seedling development but provided no significant data on phytaspase activity in the roots or shoots. Thus, it appears that drought, salt stress, and heat stress do not engage phytaspase in molecular pathways that form long-term responses to stress. For better comprehension, we have grouped the obtained data for all types of stress into a summary—Table 1.

Collectively, these data suggest that phytaspase activity is selectively induced by specific stressors, primarily mechanical wounding, whereas the drought, heat, and salinity stress molecular responses do not involve pathways regulating phytaspase gene expression. The variability in responses emphasizes the need for a deeper understanding of phytaspase regulation, including transcriptional, post-transcriptional, and post-translational mechanisms under diverse conditions. Future research should focus on detailed temporal sampling, broader stress gradients, and complementary molecular analyses, potentially involving plants with overexpressed or silenced phytaspase genes. Elucidating phytaspase’s role in stress and adaptive responses will advance strategies for enhancing plant resilience.

Although the mechanisms of phytaspase activation and its intracellular targets during long-term stress responses remain unexplored, the observed selective induction suggests its involvement in JA-related signaling rather than SA-mediated pathways. Mechanical wounding, typically associated with JA signaling, activates phytaspase, while abiotic stresses such as heat and salinity, where SA may play a more prominent role, do not. This may point to a regulatory divergence or inhibitory crosstalk between the JA and SA pathways, as previously described in other stress models.

Moreover, mechanical wounding strongly induces Ca^2+^ influx, one of the earliest events in plant stress signaling. The plausible involvement of phytaspase in calcium signaling is supported by its apoplastic localization and potential interaction with calcium-binding proteins such as calreticulin. Since calreticulin trafficking is thought to be affected by the phytaspase-mediated removal of the ER retention signal [45], it is conceivable that this proteolytic event may influence calcium signaling at the early stages, potentially by altering calreticulin availability or distribution during the initial mechanical stress response. This hypothesis warrants further investigation and could uncover novel molecular links between calcium dynamics and protease-mediated stress responses.

## 4. Materials and Methods

### 4.1. Mature Plant Growth Conditions

*N. benthamiana* wild-type plants were grown in 10 × 10 × 14 cm pots filled with special vegetable soil, a standard commercial potting mix for vegetables (Stanley Agricultural Group, Linyi, China). The water holding capacity (WHC) of this substrate was determined to be approximately 51 ± 4% using a simple saturation method [55]. The plants were grown in a controlled environment under a 16 h light (23 °C)/8 h dark (21 °C) cycle. Light intensity was 10,000 Lx, provided by full-spectrum white LEDs, which corresponds to approximately 200 μmol/m^2^/s PPFD, in an artificial climate chamber (Ningbo Southeast Instrument, Ningbo, China). The CO_2_ level was 400 ± 20 ppm during the light period and 540 ± 20 ppm during the dark period (HT-2000, Dongguan Xintai Instrument, Dongguan, China). The 4–6-week-old plants were used for experiments, and the age of plants is marked in corresponding sections.

### 4.2. Wounding Stress

For the mildest wounding, we lightly mechanically disrupted the leaf lamina as described in [52]. For other wounding conditions, we removed a specific number of mature leaves at the petiole level. The experiments were conducted with three biological replicates. Each experiment specifies whether a control group of untreated plants of the same age and comparable physiological condition was used, or whether samples collected from the experimental group on Day 0 served as an internal control. The plants were maintained in a climatic chamber under controlled conditions throughout the experiment.

### 4.3. Drought Stress

To induce drought stress, we selected three 4-week-old *N. benthamiana* plants and withheld watering. A control group of three age-matched plants was maintained under standard watering conditions. All plants were grown in a climate chamber under controlled conditions, including 60% relative humidity. After 20 days without watering, the experimental group exhibited initial wilting symptoms. From Day 20 onward, we added 10 mL of water to each pot in the experimental group every 3 days. Soil moisture was measured using a soil moisture tester (Wuhan Jingyuyuan Tech., Wuhan, China) according to the manufacturer’s instructions.

On Day 34, the wilting symptoms became too severe, and we terminated the drought experiment, switching to abundant watering followed by standard watering for the experimental group, designated as the recovery period.

To detect the molecular markers of drought stress, free proline levels were quantified in leaf tissues using a modified colorimetric assay [56]. Ninhydrin reagent was freshly prepared by dissolving 0.125 g ninhydrin (>98%, Macklin, Shanghai, China) in 3 mL glacial acetic acid (>99.5%, Foshan Xilong Chemical, Foshan, China), 2 mL of 6 M phosphoric acid (>85%, Foshan Xilong Chemical, Foshan, China), and distilled water to a final volume of 10 mL. The leaf samples were homogenized in 3% acetic acid directly in reaction tubes using a micro-pestle, then heated at 65 °C for 5 min with intermittent mixing. After centrifugation (10 min at 12,000× *g*), 200 μL of the supernatant was incubated with 200 μL of ninhydrin reagent in a water bath at 95 °C for 30 min, followed by the addition of 400 μL distilled water. The proline concentration was determined spectrophotometrically by measuring absorbance at 514 nm in a 96-well transparent flat-bottom plate using a Spark multifunctional microplate reader (Tecan Austria GmbH, Grödig, Austria). The pellet was subsequently dried to determine dry weight, and proline levels were expressed relative to tissue dry mass.

### 4.4. Overheating

To induce heat stress, we used a thermostat for short-term exposure and a climate chamber with elevated temperatures, different from those in Section 4.1, for long-term exposure. Specific temperatures are detailed in the corresponding results. In the seedling experiments, *N. benthamiana* seeds were stratified for 3 days at 4 °C and then sown in six Petri dishes containing standard potting soil. The experimental group (three dishes) was placed under heat stress conditions, while the control group (three dishes) was maintained in a climate chamber under standard conditions as described in Section 4.1. After 2 weeks, when seedlings fully emerged, we measured phytaspase activity and phytaspase gene expression in samples from both the experimental and control groups.

### 4.5. Salinity Stress

*N. benthamiana* seeds were sterilized with 70% ethanol (Shanghai Lingfeng Chemical Reagent, Shanghai, China) for 1 min, followed by 7% sodium hypochlorite (Aladdin, Shanghai, China) with 0.05% Tween 20 (>99%, Macklin, Shanghai, China) treatment for 10 min, and rinsed thrice with distilled water. Seeds were stratified at 4 °C for 2 days, then sown on square Petri dishes containing half-strength MS under the following conditions: 2.2 g/L Murashige and Skoog salts (Qingdao Hope, Qingdao, China), 10 g/L glucose (>99%, Shanghai Lingfeng Chemical Reagent, Shanghai, China), 0.5 g/L MES (>99%, Macklin, Shanghai, China), 8 g/L agar (>99%, Guandong Huankai Microbial, Guangzhou, China), pH 5.7, with or without 100 mM NaCl (>99.5%, Shanghai Lingfeng Chemical Reagent, Shanghai, China). The seedlings were grown for 14 days in a controlled environment with a 16 h day at 23 °C and an 8 h night at 21 °C in an artificial climate chamber using a vertical Petri dish orientation. Root length was measured every 2 days until day 14, and roots and shoots were then collected separately for biomass weighing and phytaspase activity assay.

### 4.6. Phytaspase Activity Assay

To detect phytaspase activity, the fluorogenic peptide substrate Ac-VEID-AFC (Aladdin, Shanghai, China) was used, previously identified as the optimal substrate for *N. benthamiana* phytaspase NbSBT1.9-2 [51]. The reaction mixture contained 10 μM Ac-VEID-AFC, B1 buffer, 500 mM NaCl, and an aliquot of the sample. The B1 buffer contained 50 mM MES, 2 mM DTT (>99%, Macklin, Shanghai, China), 0.1% Tween 20 (for molecular biology, Macklin, Shanghai, China), 5% glycerol (>99%, Shanghai Lingfeng Chemical Reagent, Shanghai, China), and 50 mM NaCl, with a pH of 5.5 adjusted using NaOH (>96%, Macklin, Shanghai, China). For sample preparation, 9 parts of B1 buffer supplemented with a protease inhibitor cocktail of 6 μg/mL leupeptin (Macklin, Shanghai, China), 2 μg/mL aprotenin (Macklin, Shanghai, China), 25 μg/mL AEBSF (Sigma, St. Louis, MO, USA), and 5 μg/mL E64 (Sigma, St. Louis, MO, USA) were added to 1 part of plant material (*v*/*w*), which was ground with a pestle and centrifuged at 14,000 × *g* for 10 min. For the phytaspase activity detection, 5 μL of supernatant per well was used. Fluorescence intensity was measured in 50 μL of the reaction mixture in a 96-well dark-walled flat-bottom plate using a Spark multifunctional microplate reader (Tecan Austria GmbH, Grödig, Austria) every 10 min for 4 h at an excitation wavelength of 400 nm and an emission wavelength of 505 nm at 28 °C. The increase in fluorescence intensity per hour was calculated as the rate of phytaspase reaction with the substrate. For statistical analysis, each sample was prepared in 3 biological repeats, and a *t*-test was performed to determine *p*-values.

### 4.7. RNA Purification and qPCR

Total RNA samples were obtained from 30 to 50 mg of plant material using the NucleoSpin RNA Plant kit (Makherey-Nagel, Düren, Germany), reverse transcribed using RevertAid reverse transcriptase (Thermo Scientific, Waltham, MA, USA), and used to determine the relative levels of NbSBT1.9-2 mRNA. Real-time qPCR reactions were performed with a Power qPCR mixture Premix (Generay Biotech, Shanghai, China) and NbSBT1.9-2_qPCR_dir (5′-TCAAGCGAAAGCTCC-3′), NbSBT1.9-2_qPCR_rev (5′-GTTATAAAGACAGCC-3′) primers using the C1000 Thermal Cycler equipped with the CFX96 Real-Time System (Bio-Rad Laboratories, Hercules, CA, USA). The 2^−ΔΔCt^ method was used to calculate relative gene expression changes, with *NbEF1a* or *Nb18S* rRNA as reference genes [57]. Optimal amplification efficiency for *NbSBT1.9-2* was achieved by adding 5% glycerol (>99.5%, Sigma-Aldrich, St. Louis, MO, USA).

### 4.8. Statistical Analysis

Statistical evaluation was performed using Fisher’s test for homogeneity of variances, followed by two-tailed Student’s *t*-test to assess the significance between experimental groups. Thresholds of *p* < 0.05, *p* < 0.01, and *p* < 0.001 were considered statistically significant. Experiments were performed on three individual plants per condition (e.g., control, mild wounding, severe wounding). Key experiments showing notable or unclear effects, such as selected wounding intensities and drought treatments, were reproduced across independent biological batches to confirm reproducibility. In seedling-based experiments, each biological sample represented pooled tissue from several individuals to ensure sufficient biomass for reliable measurements. Data are presented as mean ± SD unless otherwise stated. Cases with coefficient of variation (CV) exceeding 20% were marked as highly variable and interpreted with caution. Statistical analyses were performed using Microsoft Excel (Version 2506, Microsoft 365, Microsoft Corporation, Redmond, WA, USA).

## 5. Conclusions

Our data suggest that phytaspase activity is selectively induced by specific stressors, such as mechanical wounding, whereas drought, heat, and salt stress likely do not involve phytaspase-related pathways. The variability in responses emphasizes the need for a deeper understanding of phytaspase regulation, encompassing its transcriptional, post-transcriptional, and post-translational control under diverse conditions. Future studies should focus on detailed temporal sampling, broader stress gradients, and additional molecular analyses to elucidate the role of phytaspase in plant stress responses, potentially using plants overexpressing phytaspase or with reduced enzyme activity. Understanding phytaspase involvement in stress and adaptive responses will enhance the opportunities for improving plant resilience.

## Figures and Tables

**Table 1:** Phytaspase activity and gene expression under various stress conditions.

Section	Treatment	Phytaspase Activity (Fold Change)	Phytaspase GeneExpression (Fold Change)
2.1	Mild lamina damage of 1 leaf (4-week-old plants, Day 14)	0.93 ± 0.12, *p* = 0.22	-
2.4	Removal of 2 leaves (5-week-old plants, Day 9)	1.02 ± 0.21, *p* = 0.80	-
2.4	Removal of 3 leaves (5-week-old plants, Day 9)	1.44 ± 0.36 *	-
2.1	Removal of 4 leaves (4-week-old plants, Day 14)	1.47 ± 0.28 *	-
2.2	Removal of 5 leaves, single-point sampling (6-week-old plants, Day 9)	1.62 ± 0.60 *	1.28 ± 0.26 *
2.2	Removal of 5 leaves, sequential sampling (6-week-old plants)		
	Day 9	2.23 ± 0.29 **	-
	Day 15	2.38 ± 0.37 **	-
	Day 21	2.52 ± 0.46 ***	-
2.3	Removal of 6 leaves, repeated severe wounding (7-week-old plants, 21 days after first removal of 4 leaves)		
	Day 6	1.23 ± 0.12 **	11.22 ± 3.12 **
	Day 9	1.70 ± 0.15 ***	7.29 ± 2.91 *
2.5	Drought (no watering, 4-week-old plants, Day 34)	0.84 ± 0.54, *p* = 0.55	1.03 ± 1.17, *p* = 0.43
2.6	Heat stress (37 °C overnight, 4-week-old plants)	1.13 ± 0.18, *p* = 0.18	-
2.6	Heat stress (30 °C for 5 days, 4-week-old plants)	0.95 ± 0.22, *p* = 0.66	-
2.6	Heat stress (27 °C for 2 weeks, seedlings, Day 14)	0.79 ± 0.36, *p* = 0.27	-
2.7	Salinity stress (100 mM NaCl, seedlings, Day 14)		
	Roots	1.09 ± 0.12, *p* = 0.22	-
	Shoots	0.77 ± 0.39, *p* = 0.34	-

All values represent mean ± SD (n = 3 biological replicates). Dash (-) indicates data not determined. Asterisks indicate statistical significance versus control: * *p* < 0.05, ** *p* < 0.01, *** *p* < 0.001.

## Data Availability

Data available on osf.io by http://doi.org/10.17605/OSF.IO/HKEJP.
